# Overlap of Antibiotic Resistant *Campylobacter jejuni* MLST Genotypes Isolated From Humans, Broiler Products, Dairy Cattle and Wild Birds in Lithuania

**DOI:** 10.3389/fmicb.2019.01377

**Published:** 2019-06-19

**Authors:** Jurgita Aksomaitiene, Sigita Ramonaite, Egle Tamuleviciene, Aleksandr Novoslavskij, Thomas Alter, Mindaugas Malakauskas

**Affiliations:** ^1^Department of Food Safety and Quality, Faculty of Veterinary Medicine, Veterinary Academy, Lithuanian University of Health Sciences, Kaunas, Lithuania; ^2^Clinic of Children Diseases, Medical Academy, Lithuanian University of Health Sciences, Kaunas, Lithuania; ^3^Institute of Food Safety and Food Hygiene, Freie Universität Berlin, Berlin, Germany

**Keywords:** *Campylobacter jejuni*, MLST, antimicrobial resistance, multidrug resistance, humans, broiler, cattle, wild birds

## Abstract

Antimicrobial resistance was determined for 341 thermophilic *Campylobacter jejuni* isolates obtained from human clinical cases (*n* = 101), broiler products (*n* = 98), dairy cattle (*n* = 41) and wild birds (*n* = 101) with known multilocus sequence types (MLST) in Lithuania. The minimum inhibitory concentration (MIC) values for ciprofloxacin, tetracycline, gentamicin, ceftriaxone and erythromycin were determined with the agar dilution method. MIC values were compared with MLST types to find possible associations among isolation source, sequence type and resistance to antibiotics. The proportions of resistant strains were 94.2% (human), 95% (wild birds), 100% (broiler products) and 100% (dairy cattle) for one of the tested antibiotics. Most frequently, resistance to ciprofloxacin was observed (91.5%), followed by ceftriaxone with 60.4%, and tetracycline (37.8%). However only three *C. jejuni* strains were resistant to erythromycin (0.9%) and all tested thermophilic *Campylobacter* strains were sensitive to gentamicin. Most of the examined *C. jejuni* isolates (80.6%) showed resistance to at least one of three profiles: CIP+AXO (28.1%), TET+CIP+AXO (26.7%) and CIP (25.8%). Statistically significant differences in resistance to tetracycline were found between *C. jejuni* strains obtained from cattle (85.4%) and broiler products (64.3%) (*P* < 0.05). The majority (87.1%) of the tested strains from wild birds were resistant to ciprofloxacin (*P* < 0.05). The results showed that strains of novel ST’s showed significantly lower resistance to ceftriaxone (*P* < 0.05). The ST-21 (CC21) (78.8%) was identified with significantly higher multidrug resistance relatively to other tested ST’s in this study. Our results emphasize the high antimicrobial resistance of phylogenetically diverse *C. jejuni* strains isolated from different sources including specific genotypes of wild bird’s strains in Lithuania. The results support the opinion that not only broiler products but cattle and wild birds may be a reservoir of resistant *C. jejuni* and stipulate a risk of spread or resistant bacteria. There is increasing need for broad surveillance and control measures to track changes and pathways of antimicrobial resistance of *C. jejuni* in epidemiologically distinct populations.

## Introduction

*Campylobacter* spp. are zoonotic pathogens and a main cause of human bacterial intestinal disease worldwide. Thermophilic *Campylobacter* caused infection is one of the most commonly reported foodborne zoonosis in the European Union (EU) since 2005. The number of reported confirmed cases of human campylobacteriosis was 246,307, with an EU notification rate of 66.3 per 100,000 population (World Health Organization [WHO], 2013; EFSA and ECDC, 2017). This foodborne infection is primarily associated with consumption of poultry products, followed by cattle associated food products (French et al., 2009; de Haan et al., 2010; Mughini Gras et al., 2012). The majority of foodborne infections caused by thermophilic *Campylobacter* are mild and generally self-limited within a few days without antimicrobial treatment. However, complications can arise and may include bacteremia, Guillain-Barre syndrome, reactive arthritis, and abortion (Skirrow and Blaser, 2000). Usually, erythromycin (or other macrolides), ciprofloxacin (fluoroquinolone) are used for the treatment of human campylobacteriosis (Blaser and Engberg, 2008). Fluoroquinolones are used widely for empirical treatment for enteroinvasive bacterial diarrhea meanwhile the third-generation cephalosporin’s like a ceftriaxone or gentamicin (aminoglycoside) – for serious systematic infections. The choice is based on the effective treatment option for common human *Campylobacter* infection (Nahata and Barson, 1985; Pacanowski et al., 2008; Ge et al., 2013). However, due to the fact that in some countries extremely high acquired resistance to fluoroquinolones is detected, it can no longer be considered for empirical treatment of human *Campylobacter* infection (EFSA and ECDC, 2018). Although tetracyclines are not often used in practice, sometimes these antimicrobials could be considered as an alternative choice in the therapy of *Campylobacter* infection (Luangtongkum et al., 2009; Wieczorek and Osek, 2013). However, thermophilic *Campylobacter* is becoming increasingly resistant to antimicrobials used in clinical practices, and the increasing resistance of these bacteria may have an adverse effect on public health. Application of large amounts of antibiotics for human therapy as well as for farm animals resulted in the selection of pathogenic bacteria including thermophilic *Campylobacter* resistant to multiple drugs (McDermott et al., 2002; Luangtongkum et al., 2009; Juntunen et al., 2011). The increasing resistance to antibiotics, particularly the high level of ciprofloxacin-resistant thermophilic *Campylobacter* strains in broilers, is a concern also in the EU (EFSA and ECDC, 2018).

The antimicrobial resistance of thermophilic *Campylobacter* strains can be associated with specific isolation sources of these bacteria. Thus, the high rate of resistant thermophilic *Campylobacter* strains from wild birds indicate that this source might be an important vector for the distribution of resistant *Campylobacter* and the potential transfer to humans, cattle and other sources.

The aim of this study was to investigate the phenotypic antimicrobial resistance of *C. jejuni* strains in association with MLST genotypes and isolation source (strains from human clinical cases, broiler products, dairy cattle and wild birds) in Lithuania.

## Materials and Methods

### *C. jejuni* Strains

A total of 341 *C. jejuni* strains of known Multilocus sequence types (MLST) were included in this study. The strain collection was composed of 41 strains isolated from dairy cattle at farm level, 98 strains isolated from retail broiler products, 101 strains isolated from wild birds and 101 strains from human clinical cases that were collected at the Microbiological Laboratory of Kaunas Clinical Hospital over one year period. The MLST method based on sequencing of seven housekeeping genes was applied for each of the isolates as described in a previous study (Ramonaite et al., 2014, 2017). The strains were stored at -80°C in brain heart infusion broth (BHI) (Oxoid, Basingstoke, United Kingdom) with 30 % glycerol (Stanlab, Lublin, Poland).

### Detection of Minimum Inhibitory Concentration (MIC)

The *C. jejuni* strains were tested against phenotypic resistance to five antimicrobial agents (ciprofloxacin, CIP, tetracycline, TET, gentamicin, GEN, ceftriaxone, AXO and erythromycin, ERY), (all Sigma-Aldrich, Saint-Louis, MO, United States) by the agar dilution method according the Clinical and Laboratory Standards Institute (CLSI) guidelines (CLSI, 2006). Mueller-Hinton agar (Oxoid) with dilutions ranging from 0.25 to 256 mg/L for ciprofloxacin, tetracycline, gentamicin, ceftriaxone and erythromycin was prepared. For each sample, 5 μl of approximately 1×10^7^ CFU/ml (*OD*_600_ = 0.1) bacterial suspension dissolved in PBS (phosphate-buffered saline, Oxoid) was spotted onto Mueller-Hinton agar containing the corresponding antimicrobial agent and incubated at 37°C for 48 h. The experiment for all isolates was performed in triplicate. The MIC values were defined as the lowest concentration that produces complete inhibition of *C. jejuni* growth. For quality control, the reference strain *C. jejuni* NCTC 11168 was included. Following MIC interpretive criteria for resistance were used: erythromycin (≥32), tetracycline (≥16), ciprofloxacin (≥4), gentamicin (≥16) and ceftriaxone (≥16) (CLSI, 2006). Isolates showing resistance to three or more groups of antimicrobials were considered as multidrug resistant (MDR).

### Statistical Analysis

The statistical package SPSS (Statistics 20, IBM, Armonk, NY, United States) was used for data processing and statistical analysis. Chi-square test was used to test for statistically significant associations between resistance to different antimicrobial drugs and between resistance and MLST type. A *P* value of <0.05 was used to indicate statistically significant results.

## Results

### Genetic Characterization of Antimicrobial Resistance

**FIGURE 1:**
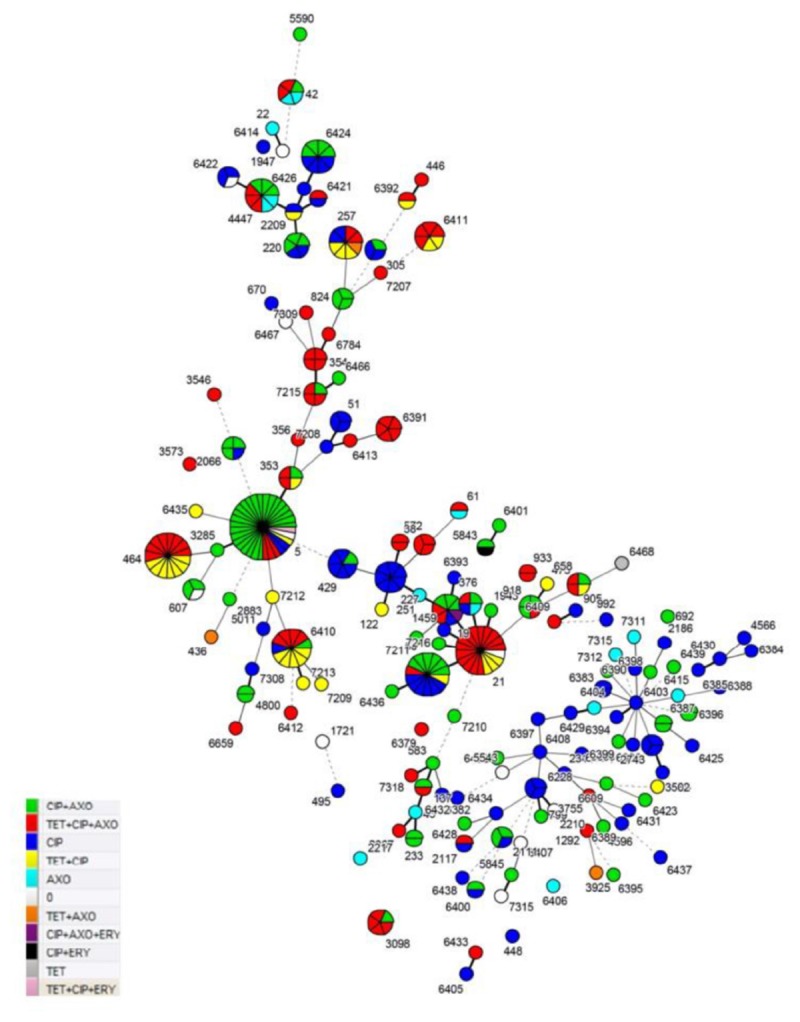
MLST profile and resistance profiles of *C. jejuni* sequence types (STs) from human clinical cases, broiler products, dairy cattle and wild birds from Lithuania (Detailed information on these MLST types is provided in Supplementary Table S2).

Resistance to ciprofloxacin, tetracycline and ceftriaxone was found in 91.5, 37.8, and 60.4%, of the tested *C. jejuni* strains, respectively. Only three strains were resistant to erythromycin with a MIC of 32 μg/mL (0.9%) and two of them were isolated from humans (ST-5; ST-19) and one from wild birds (ST-5843), respectively. Meanwhile, all *C. jejuni* strains were sensitive to gentamicin. We identified ten *C. jejuni* specific antimicrobial resistance profiles (Table 1) and most of the examined *C. jejuni* strains (80.6%) showed resistance to one of three profiles: CIP+AXO (28.1%), TET+CIP+AXO (26.7%) and CIP (25.8%). The TET+CIP+AXO multidrug resistance profile was confirmed for the majority of the examined *C. jejuni* strains assigned to ST-21 (77.8%), ST-3098 (80%), ST-354 (100%), ST-464 (53.3%), ST-6411 (66.7%), and ST-6391 (100%) Most of the examined *C. jejuni* isolates which belonged to the ST-5 (75%) were confirmed as CIP+AXO profile resistant (Figure 1 and Supplementary Table S2).

**TABLE 1:** Antimicrobial resistance profiles of *C. jejuni* strains isolated from different sources.

	**Source of isolate and resistance rating**									
**Antimicrobial resistance profiles**	**Human cases**		**Broiler products**		**Dairy cattle**		**Wild birds**		**All tested sources**	
	**N**	**%**	**N**	**%**	**N**	**%**	**N**	**%**	**N**	**%**
CIP+AXO	34	33.7	27	27.6	4	9.7	31	30.7	96	28.1
TET+CIP+AXO	8	7.9	42	42.9	33	80.5	8	7.9	91	26.7
CIP	33	32.7	7	7.1	–	–	48	47.5	88	25.8
TET+CIP	12	11.9	21	21.4	–	–	–	–	33	9.7
AXO	5	5	1	1	2	4.9	7	6.9	15	4.4
TET+AXO	–	–	–	–	2	4.9	1	1	3	0.9
TET+CIP+ERY	1	1	–	–	–	–	–	–	1	0.3
CIP+AXO+ERY	1	1	–	–	–	–	–	–	1	0.3
CIP+ERY	–	–	–	–	–	–	1	1	1	0.3
TET	1	1	–	–	–	–	–	–	1	0.3
Total resistance	95	94.2	98	100	41	100	96	95	330	96.8
Sensitive	6	5.8					5	5	11	3.2

*TET, tetracycline; ERY, erythromycin; CIP, ciprofloxacin; GEN, gentamicin; AXO, ceftriaxone.*

Of the 341 tested *C. jejuni* strains, 330 (96.8%) were resistant to at least one of the tested antimicrobials, whereas 11 strains (3.2%) were sensitive to all five tested antibiotics. A total of 133 strains (39.0%) were resistant to two different classes of the tested antibiotics and 93 strains (27.3%) were resistant to three classes of antibiotics and were considered as multidrug resistant (MDR) (Table 1).

All *C. jejuni* strains resistant to ciprofloxacin and ceftriaxone showed high MICs (4-256, 16-128 μg/mL, respectively). A significant percentage (51.1%) of tetracycline resistant strains isolated from human clinical cases (77.3%), broiler products (42.9%) and wild birds (44.5%) displayed a high-level resistance with MIC values in the range from 64 up to 256 μg/mL (Table 2).

**TABLE 2:** Minimum inhibitory concentration values for *C. jejuni* strains isolated from different sources against antimicrobial agents.

**Sources**	**Antibiotic**	**No. of sensitive strains/%**	**No. of resistant strains/%**	**Antimicrobial agents MIC for *C. jejuni* isolates (MIC; μg/mL)**										
				**0.25**	**0.5**	**1**	**2**	**4**	**8**	**16**	**32**	**64**	**128**	**256**
**Human cases (*N* = 101)**	TET	79/78.2	22/21.8		41/40.6	32/31.7	5/5.0		1/1.0		5/5.0	10/9.9	4/4.0	3/3.0
	ERY	99/98.0	2/2.0		16/15.8	54/53.5	28/27.7	1/1.0			2/2.0			
	CIP	12/11.9	89/88.1			2/2.0	10/9.9		3/3.0	40/39.6	27/26.7	17/16.8	2/2.0	
	GEN	101/100	0/0	21/20.8	69/68.3	8/7.9		3/3.0						
	AXO	53/52.5	48/47.5	1/1.0		1/1.0	3/3.0	13/12.9	35/34.7	38/37.6	7/6.9	2/2.0	1/1.0	
**Broiler products (*N* = 98)**	TET	35/35.7	63/64.3		13/13.3	6/6.1	6/6.1	7/7.1	3/3.1	11/11.2	25/25.5	9/9.2	11/11.2	7/7.1
	ERY	98/100	0/0	18/18.4	26/26.5	32/32.7	19/19.4	3/3.1						
	CIP	0/0	98/100					5/5.1	11/11.2	33/33.7	12/12.2	23/23.5	13/13.3	1/1.0
	GEN	98/100	0/0	36/36.7	41/41.8	20/20.4	1/1.0							
	AXO	28/28.6	70/71.4				1/1.0	3/3.1	24/24.5	32/32.7	21/21.4	14/14.3	3/3.1	
**Dairy cattle (*N* = 41)**	TET	6/14.6	35/85.4		4/9.8		1/2.4		1/2.4	3/7.3	6/14.6	22/53.7	4/9.8	
	ERY	41/100	0/0	1/2.4	11/26.8	15/36.6	7/17.1	5/12.2	1/2.4	1/2.4				
	CIP	4/9.8	37/90.2				4/9.8	2/4.9	6/14.6	18/43.9	7/17.1	1/2.4	3/7.3	
	GEN	41/100	0/0	18/43.9	21/51.2	2/4.9								
	AXO	0/0	41/100							20/48.8	15/36.6	4/9.8	2/4.9	
**Wild birds (*N* = 101)**	TET	92/91.1	9/8.9		44/43.6	6/5.9	25/24.8	9/8.9	8/7.9	1/1.0	4/4.0	1/1.0	3/3.0	
	ERY	100/99.0	1/1.0	51/50.5	11/10.9	32/31.7	3/3.0	3/3.0			1/1.0			
	CIP	13/12.9	88/87.1				13/12.9	47/46.5	27/26.7	12/11.9		2/2.0		
	GEN	101/100	0/0	36/35.6	42/41.6	20/19.8	2/2.0		1/1.0					
	AXO	54/53.5	47/46.5	1/1.0			11/10.9	6/5.9	36/35.6	19/18.8	15/14.9	11/10.9	2/2.0	
**All sources (*N* = 341)**	TET	212/62.2	129/37.8		102/29.9	44/12.9	37/10.9	16/4.7	13/3.8	15/4.4	40/11.7	42/12.3	22/6.5	10/2.9
	ERY	338/99.1	3/0.9	70/20.5	64/18.8	133/39.0	57/16.7	12/3.5	1/0.3	1/0.3	3/0.9			
	CIP	29/8.5	312/91.5			2/0.6	27/7.9	54/15.8	47/13.8	103/30.2	46/13.5	43/12.6	18/5.3	1/0.3
	GEN	341/100	0/0	111/32.6	173/50.7	50/14.7	3/0.9	3/0.9	1/0.3					
	AXO	135/39.6	206/60.4	2/0.6		1/0.3	15/4.4	22/6.5	95/27.9	109/32.0	58/17.0	31/9.1	8/2.3	

*TET, tetracycline; ERY, erythromycin; CIP, ciprofloxacin; GEN, gentamicin; AXO, ceftriaxone; MIC, minimum inhibitory concentration.*

Among the 341 *C. jejuni* strains included in the antimicrobial testing and MLST analysis, 146 distinct sequence types (STs) were identified. Theses STs were assigned to 26 previously described clonal complexes (CCs). In this study, 231 of *C. jejuni* strains were included, which were grouped into previously known sequence types (ST) and 110 isolates, which were assigned to novel STs in a previous study (Ramonaite et al., 2014, 2017). There were 110 (32.3%) *C. jejuni* strains representing 79 (54.1%) STs that were previously described by our group as new STs. Clonal complexes CC21 [50 (14.7%) strains], CC353 [42 (12.3%) strains] and CC179 [26 (7.6%) strains] were dominant among *C. jejuni* found among all tested sources and accounted for 34.6% of all strains. All ST-464 (CC464) *C. jejuni* strains were resistant to tetracycline and ciprofloxacin (*P* < 0.05). This ST was dominant among *C. jejuni* strains isolated from broiler products. ST-5 (CC353) was dominant among *C. jejuni* strains isolated from human clinical cases and 96.9% of these isolates were resistance to ciprofloxacin and 84.4% showed resistance to ceftriaxone. The results showed that strains belonged to ST-21 (CC21) (77.8%) exhibited a significantly higher multidrug resistance (*P* < 0.05). The results showed that strains belonging to ST-21 (CC21) (94.4%), ST-257 (75%), ST-3098 (80%), ST-353 (75%), and ST-354 (100%) were resistant to tetracycline. ST-21 was dominant among dairy cattle in our study. Though, some of the sequence types (ST-137, ST-2067, ST-354, ST-3546, ST-356, ST-3573, ST-3755, ST-38, ST-446, ST-572, ST-6391, ST-6409, ST-6412, ST-6413, ST-6433, ST-6784, ST-7207, ST-7309, ST-7318, and ST-933) were not dominant in our study and all strains assigned to these STs were MDR (Figure 1). Additionally, *C. jejuni* sequence types assigned to CC1034 (100%), CC354 (81.8%), CC403 (100%), CC443 (55.6%), CC446 (66.7%), CC464 (53.3%) and CC61 (50%) showed high multidrug resistance (Supplementary Table S1).

### Antimicrobial Resistance of *C. jejuni* Isolates From Human Clinical Cases

The lowest percentage of resistant *C. jejuni* strains isolated from different sources was detected in humans (94.2%) and resistance to CIP alone was the most frequently observed (88.1%). Six *C. jejuni* (5.9%) strains isolated from human clinical cases were sensitive for all tested antibiotics (Table 1). Moreover, all strains isolated from human clinical cases were sensitive to gentamicin. Only two strains (2%) were resistant to erythromycin and these strains were assigned to ST-5 (CC353) and ST-19 (CC21). Most of the examined *C. jejuni* strains from human clinical cases showed resistance to one of two profiles: CIP+AXO (33.7%) and CIP (32.7%). The ST-5 and ST-50, which are linked to CC353 and CC21, were predominant among the *C. jejuni* strains isolated from human clinical cases. ST-50 strains showed a 100% resistance to ciprofloxacin and 100% were sensitive to erythromycin and gentamicin. ST-5 strains showed a 95.7% resistance to ciprofloxacin (Supplementary Tables S1, S2).

### Antimicrobial Resistance of *C. jejuni* Isolates From Broiler Products

All *C. jejuni* strains from broiler products were resistant to at least one of our tested antibiotics. In total, 100% of the tested strains were resistant to CIP. In addition, a high number (71.4%) of ceftriaxone resistance strains was detected. All *C. jejuni* strains from broiler products were sensitive to erythromycin and gentamicin (Table 2). Most of the examined *C. jejuni* strains isolated from broiler products showed resistance to TET+CIP+AXO (42.9%) (Table 1). Three STs (ST-5, ST-464 and ST-6410) were the most prevalent among *C. jejuni* strains detected in broiler products. ST-464 strains were 100% and ST-6410 strains were 81.8% resistant to tetracycline. ST-5, ST-464 and ST-6410 were 100% resistant to ciprofloxacin. ST-5 was 100% resistant to ceftriaxone (Supplementary Table S1).

### Antimicrobial Resistance of *C. jejuni* Isolates From Dairy Cattle

All *C. jejuni* strains from dairy cattle were resistant to at least one of our tested antibiotics. Ceftriaxone, ciprofloxacin and tetracycline resistance was detected in 100, 90.2, and 85.4% of the tested dairy cattle strains, respectively. All *C. jejuni* strains isolated from dairy cattle were sensitive to erythromycin and gentamicin (Table 2). Most of the examined *C. jejuni* strains from dairy cattle showed resistance to TET+CIP+AXO (80.5%) (Table 1). CC21 and ST-21 were predominant among *C. jejuni* strains detected in dairy cattle. All ST-21 strains showed multidrug resistance to TET+CIP+AXO (Supplementary Table S1).

### Antimicrobial Resistance of *C. jejuni* Isolates From Wild Birds

Altogether, 95% of the *C. jejuni* strains from wild birds were resistant to at least one of the five tested antibiotics. The majority of the tested strains (87.1%) were resistant to ciprofloxacin (Table 2). Only one *C. jejuni* strain, isolated from wild birds, was resistant to erythromycin and this strain was assigned to ST-5843. All *C. jejuni* strains isolated from wild birds were sensitive to gentamicin. Most of the examined *C. jejuni* strains isolated from wild birds showed resistance to one of two profiles: CIP+AXO (30.7%) and CIP (47.5%) (Table 1). *C. jejuni* strains from wild birds showed lower multidrug resistance (7.9%) and also lower resistance to tetracycline (8.9%) in comparison to *C. jejuni* isolated from dairy cattle and broiler products isolates.

## Discussion

Antimicrobial resistance, particularly multidrug resistance, is an emerging and essential public health problem (Van Looveren et al., 2001; Noormohamed and Fakhr, 2014). Zoonotic bacteria that are resistant to antimicrobials are of particular concern, as they might compromise the effective treatment of infections in humans. This enabled analysis of multidrug resistance (MDR) and co-resistance patterns to critically important antimicrobials in both human and animal isolates at the EU level but also at country level. Erythromycin resistance in thermophilic *Campylobacter* is of public health significance, but occurs at low, very low or undetected levels in many European countries. Based on the ECDC report, the level of resistance to erythromycin was overall relatively low (at 1.5%), but varied between countries (ECDC, 2017). The low number (0,9%) of erythromycin resistant *C. jejuni* strains found in our study corresponds to reports from other countries such as Finland (1,1%) (Lehtopolku et al., 2010), Italy (1,4%) (Pezzotti et al., 2003) and Japan (0,6%) (Tadano et al., 1996). Our results confirm that the level of erythromycin resistance in Lithuania is low and stable and currently erythromycin can be regarded as suitable drug for the treatment of *Campylobacter* disease.

An increase in tetracycline resistance of *C. jejuni* strains has been observed in recent years (Abdi-Hachesoo et al., 2014; .ECDC, 2017). The number of tetracycline resistant *C. jejuni* strains (37.8%) -irrespective of the source of isolation- found in our study was at a similar level as in other countries (Bywater, 2004; Gupta et al., 2004). In our study, a high-level tetracycline resistance was found in *C. jejuni* strains isolated from cattle (85.4%) and poultry products (64.3%) and this resistance was significantly higher (*P* < 0.05) than resistance in *C. jejuni* isolated from humans and wild birds. High rates of tetracycline resistance might be due to the usage of this antibiotic as a therapeutic agent in dairy cattle and poultry farms in our country (Webb et al., 2018).

A high percentage of antimicrobial resistance (60.4%) of *C. jejuni* to ceftriaxone was observed in our study. Interestingly, those strains from wild birds showed a high percentage (46.5%) of resistance to ceftriaxone, although wild birds do not have contact with antimicrobial drugs. These findings suggest that the resistance could be acquired by horizontal gene transfer or it can also be related to migration of birds (Sjölund et al., 2008). According to Auwera and Scorneaux (1985) and Hakanen et al. (2003) *C. jejuni* is not susceptible to most cephalosporins, due to alterations in the membrane structure or in porin proteins and the efflux pump system can cause resistance to this antimicrobial group (Lachance et al., 1991).

All *C. jejuni* strains were sensitive gentamicin in our study. Aminoglycosides are effective in the treatment of systemic thermophilic *Campylobacter* infections and in most studies, resistance to gentamicin has also not been observed (Sáenz et al., 2000).

Multidrug resistance of thermophilic *Campylobacter* strains was defined as resistance to at least three different antimicrobial classes (Magiorakos et al., 2012). We found that 27.3% of all tested *C. jejuni* strains were MDR with the predominant profile TET+CIP+AXO (26.7%). Moreover, this profile was predominant among *C. jejuni* strains isolated from dairy cattle (80.5%) and broiler products (42.9%). Additionally, we found that 237 (69.5%) of the examined strains were resistant against one or two antimicrobial agents and this percentage was higher than that reported in a study from Denmark (Andersen et al., 2006) and lower than that reported by a study in Poland (Wieczorek et al., 2012).

We found significant differences in resistance to antimicrobial agents among different thermophilic *Campylobacter* MLST genotypes. ST-21 (CC21) was predominant among *C. jejuni* strains isolated from dairy cattle in Lithuania. In total, 77.8% of the strains assigned to ST-21 (CC21) and isolated from dairy cattle were multidrug resistant (TET+CIP+AXO profile). Meanwhile, ST-50 (CC21) was most often related with human campylobacteriosis cases. Altogether, 54.5% of *C. jejuni* strains assigned to ST-50 (CC21) and isolated from human clinical cases were resistant to CIP and 27.3% to CIP and AXO. An association between CC21 and quinolone resistance has also been reported from Belgium (Habib et al., 2009) and Slovenia (Kovač et al., 2014) and resistance was observed in 66 and 95% of the tested bacterial stains, respectively. Strains of CC21, which have been associated with human and dairy cattle antimicrobial resistance was not detected in wild birds in this study.

The majority of *C. jejuni* strains from human and broiler products were assigned to CC353 and ST-5 in our study (Ragimbeau et al., 2008; Sheppard et al., 2009). In total, 75% of the *C. jejuni* strains assigned to ST-5 (CC353) and isolated from broiler products and human clinical cases were resistance to the CIP+AXO profile in our study (Iglesias-Torrens et al., 2018). ST-464 (CC464) and ST-6410 were also often assigned to *C. jejuni* strains isolated from broiler products. We found that all (100 %) of the tested strains assigned to CC464 and 97.6% of *C. jejuni* strains assigned to CC353 were resistant to ciprofloxacin (Cody et al., 2012). The same resistance of *C. jejuni* to ciprofloxacin has been reported in China (Zhang et al., 2016). Altogether, 61.5% of *C. jejuni* strains isolated from broiler products and assigned to ST-464 (CC464), 45.5 and 36.4% strains assigned to ST-6410 (CC464) were confirmed as TET+CIP+AXO, TET+CIP and TET+CIP+AXO resistant profiles.

Our study revealed an unusually high number of ciprofloxacin and ceftriaxone resistant *C. jejuni* strains isolated from humans, broiler products, dairy cattle and wild birds. We observed remarkably high resistance rates in *C. jejuni* from wild birds too, although wild birds do not undergo treatment or have direct contact with antimicrobial drugs. Interestingly, *C. jejuni* strains assigned to novel MLST genotypes showed frequent antibiotic resistance despite most of them were isolated from wild birds. In addition, our study revealed significant resistance to ciprofloxacin and tetracycline among *C. jejuni* assigned to CC464. The unequal frequency of antibiotics resistant strains between MLST clonal complexes and sequence types indicates more common occurrence of resistance determinants in some genotypes.

In conclusion, the high antimicrobial resistance of phylogenetically diverse *C. jejuni* strains isolated from different sources including specific genotypes of wild bird’s strains in Lithuania. The results of the study demonstrate that not only broiler products but also cattle and wild birds may be a reservoir for resistant *C. jejuni* and these reservoirs represent a risk to spread resistant bacteria. These results suggest the need for broad surveillance and control measures to track changes and pathways of antimicrobial resistance of *C. jejuni* in epidemiologically distinct populations.

## Author Contributions

JA, SR, AN, and MM conceived the study. ET and SR took the samples. JA and SR interpreted the data and wrote the manuscript. TA and MM provided valuable references and suggestions during the preparation of the manuscript. All authors edited the final version of the manuscript.

## Conflict of Interest Statement

The authors declare that the research was conducted in the absence of any commercial or financial relationships that could be construed as a potential conflict of interest.
