# Supplementary material for: Overlap of Antibiotic Resistant Campylobacter jejuni MLST Genotypes Isolated From Humans, Broiler Products, Dairy Cattle and Wild Birds in Lithuania
Source: Front Microbiol. 2019 Jun 19;10:1377. doi: 10.3389/fmicb.2019.01377 (PMC6593065; doi:10.3389/fmicb.2019.01377)
Supplement: Supplementary file 1 [file Table_1.DOCX]

Supplementary Material

Overlap of antibiotic resistant *Campylobacter jejuni* MLST genotypes isolated from humans, broiler products, dairy cattle and wild birds in Lithuania

Jurgita Aksomaitiene^1^*, Sigita Ramonaite^1^, Egle Tamuleviciene^2^, Aleksandr Novoslavskij^1^, Thomas Alter^3^, Mindaugas Malakauskas^1^

*** Correspondence:** Jurgita Aksomaitiene: [jurgita.aksomaitiene@lsmuni.lt](mailto:jurgita.aksomaitiene@lsmuni.lt)

# Table S1

Multidrug antimicrobial resistance of the *C. jejuni* clonal complex and sequence types obtained from various sources.

| CC | ST | Multidrug resistance | | | | Antimicrobial agents | | | |
| --- | --- | --- | --- | --- | --- | --- | --- | --- | --- |
|  |  | 0 | 1 | 2 | 3 | TET | CIP | AXO | ERY |
|  |  | No. of sensitive isolates | No. of resistance isolates to 1 antimicrobial agents | No. of resistance isolates to 2 antimicrobial agents | No. of resistance isolates to 3 antimicrobial agents |  |  |  |  |
|  | 1721 | 1(H) |  |  |  |  |  |  |  |
|  | 2117 |  | 1(WB) |  | 1(WB) | 1 | 2 | 1 |  |
|  | 2186 |  | 1(WB) |  |  |  | 1 |  |  |
|  | 2217 |  | 1(C) |  |  |  |  | 1 |  |
|  | 2743 |  | 1(WB) |  |  |  | 1 |  |  |
|  | 2883 |  |  | 1(B) |  |  | 1 | 1 |  |
|  | 3098 |  |  | 1(C) | 4(C) | 4 | 5 | 5 |  |
|  | 3502 |  |  | 1(B) |  | 1 | 1 |  |  |
|  | 3546 |  |  |  | 1(H) | 1 | 1 | 1 |  |
|  | 3573 |  |  |  | 1(B) | 1 | 1 | 1 |  |
|  | 3755 |  |  |  | 1(WB) | 1 | 1 | 1 |  |
|  | 436 |  |  | 1(C) |  | 1 |  | 1 |  |
|  | 448 |  | 1(WB) |  |  |  | 1 |  |  |
|  | 4566 |  | 1(WB) |  |  |  | 1 |  |  |
|  | 4596 |  |  | 1(WB) |  |  | 1 | 1 |  |
|  | 4800 |  |  | 1(B) |  |  | 2 | 2 |  |
|  | 495 |  | 1(H) |  |  |  | 1 |  |  |
|  | 5543 | 1(WB) |  |  |  |  |  |  |  |
|  | 5559 |  |  | 1(WB) |  |  | 1 | 1 |  |
|  | 5590 |  |  | 1(C) |  |  | 1 | 1 |  |
|  | 5843 |  |  | 2(WB) |  |  | 2 | 1 | 1 |
|  | 5845 |  |  | 1(WB) |  |  | 1 | 1 |  |
|  | 6383 |  | 1(WB) |  |  |  | 1 |  |  |
|  | 6384 |  | 1(WB) |  |  |  | 1 |  |  |
|  | 6385 |  | 1(WB) |  |  |  | 1 |  |  |
|  | 6386 |  | 3(WB) |  |  |  | 3 |  |  |
|  | 6387 |  |  | 2(WB) |  |  | 2 | 2 |  |
|  | 6388 |  | 1(WB) |  |  |  | 1 |  |  |
|  | 6389 |  | 1(WB) |  |  |  | 1 |  |  |
|  | 6390 |  | 1(WB) |  |  |  | 1 |  |  |
|  | 6394 |  | 1(WB) |  |  |  | 1 |  |  |
|  | 6395 |  |  | 1(WB) |  |  | 1 | 1 |  |
|  | 6396 |  |  | 2(WB) |  |  | 2 | 2 |  |
|  | 6398 |  |  | 1(WB) |  |  | 1 | 1 |  |
|  | 6400 |  | 1(WB) | 1(WB) |  |  | 2 | 1 |  |
|  | 6401 |  |  | 1(WB) |  |  | 1 | 1 |  |
|  | 6403 |  | 1(WB) |  |  |  | 1 |  |  |
|  | 6404 |  | 1(WB) |  |  |  |  | 1 |  |
|  | 6405 |  | 1(WB) |  |  |  | 1 |  |  |
|  | 6406 |  | 1(WB) |  |  |  |  | 1 |  |
|  | 6410 |  | 1(B) | 6(B) | 4(B) | 9 | 11 | 5 |  |
|  | 6411 |  |  | 2(B) | 4(B) | 6 | 6 | 4 |  |
|  | 6412 |  |  |  | 1(B) | 1 | 1 | 1 |  |
|  | 6415 |  | 1(WB) |  |  |  |  | 1 |  |
|  | 6422 | 1(WB) | 2(WB) |  |  |  | 2 |  |  |
|  | 6423 |  |  | 1(WB) |  |  | 1 | 1 |  |
|  | 6425 |  | 1(WB) |  |  |  | 1 |  |  |
|  | 6427 |  | 1(WB) |  |  |  | 1 |  |  |
|  | 6428 |  |  | 1(WB) |  |  | 1 | 1 |  |
|  | 6429 |  | 1(WB) |  |  |  | 1 |  |  |
|  | 6430 |  | 1(WB) |  |  |  | 1 |  |  |
|  | 6431 |  | 1(WB) |  |  |  | 1 |  |  |
|  | 6432 |  | 1(WB) |  |  |  | 1 |  |  |
|  | 6433 |  |  |  | 1(WB) | 1 | 1 | 1 |  |
|  | 6437 |  | 1(WB) |  |  |  | 1 |  |  |
|  | 6438 |  | 1(WB) |  |  |  | 1 |  |  |
|  | 6439 |  |  | 1(WB) |  |  | 1 | 1 |  |
|  | 6609 |  |  | 1(WB) |  |  | 1 | 1 |  |
|  | 7207 |  |  |  | 1(B) | 1 | 1 | 1 |  |
|  | 7209 |  |  | 1(B) |  | 1 | 1 |  |  |
|  | 7213 |  |  | 1(B) |  | 1 | 1 |  |  |
|  | 7308 |  | 1(B) |  |  |  | 1 |  |  |
|  | 7311 |  | 1(WB) |  |  |  |  | 1 |  |
|  | 7312 |  |  | 1(WB) |  |  | 1 | 1 |  |
|  | 7315 | 1(WB) | 1(WB) |  |  |  |  | 1 |  |
|  | 905 |  | 1(WB) |  |  |  | 1 |  |  |
|  | 992 |  | 1(WB) |  |  |  | 1 |  |  |
| CC1034 | 6409 |  |  |  | 1(B) | 1 | 1 | 1 |  |
| CC1275 | 3925 |  |  | 1(WB) |  | 1 |  | 1 |  |
| CC1287 | 6414 |  | 1(WB) |  |  |  | 1 |  |  |
| CC179 | 220 |  | 2(WB) | 3(WB) |  |  | 5 | 3 |  |
|  | 2209 |  | 1(H) | 1(H) |  | 1 | 2 |  |  |
|  | 4447 |  | 2(WB) | 3(WB) | 3(WB) | 3 | 6 | 8 |  |
|  | 6421 |  | 1(WB) |  | 1(WB) | 1 | 2 | 1 |  |
|  | 6424 |  | 4(WB) | 4(WB) |  |  | 8 | 4 |  |
|  | 6426 |  | 1(WB) |  |  |  | 1 |  |  |
| CC206 | 122 |  |  | 1(H) | 1 | 1 | 1 |  |  |
|  | 227 |  | 2(B);5(H) |  |  |  | 7 |  |  |
|  | 572 |  |  |  | 2(C) | 2 | 2 | 2 |  |
| CC21 | 1459 |  | 1(H) |  |  |  | 1 |  |  |
|  | 19 |  | 1(H) | 3(H) | 1(B);1(C);1(H) | 2 | 7 | 6 | 1 |
|  | 1943 |  |  | 1(H) |  |  | 1 | 1 |  |
|  | 21 | 1(H) |  | 2(B);1(H) | 14(C) | 17 | 17 | 14 |  |
|  | 251 |  | 1(B) |  |  |  | 1 | 1 |  |
|  | 376 |  | 1(B);1(H) | 1(B) | 1(B) | 1 | 3 | 3 |  |
|  | 50 |  | 6(H) | 3(B);4(H) | 1(H) | 2 | 14 | 7 |  |
|  | 6393 |  | 1(H) |  |  |  | 1 |  |  |
|  | 6436 |  |  | 1(B) |  |  | 1 | 1 |  |
|  | 7211 |  |  | 1(B) |  |  | 1 | 1 |  |
|  | 7216 |  |  | 1(C) |  |  | 1 | 1 |  |
| CC22 | 1947 | 1(H) |  |  |  |  |  |  |  |
|  | 22 |  | 1(H) |  |  |  |  | 1 |  |
| CC257 | 257 |  | 2(H) | 1(C);3(H) | 2(C) | 6 | 7 | 3 |  |
|  | 824 |  |  | 1(B);2(H) |  |  | 3 | 3 |  |
| CC283 | 6382 |  | 1(H) |  |  |  | 1 |  |  |
|  | 7210 |  |  | 1(B) |  |  | 1 | 1 |  |
| CC353 | 3285 |  |  | 1(H) |  |  | 1 | 1 |  |
|  | 353 |  |  | 2(H) | 2(B) | 3 | 4 | 3 |  |
|  | 356 |  |  |  | 1(H) | 1 | 1 | 1 |  |
|  | 5 | 1(H) | 2(H) | 6(B);19(H) | 3(B);1(H) | 5 | 31 | 27 | 1 |
|  | 5011 |  | 1(B) |  |  |  | 1 |  |  |
|  | 6413 |  |  |  | 1(B) | 1 | 1 | 1 |  |
|  | 6435 |  |  | 1(B) |  | 1 | 1 |  |  |
|  | 7212 |  |  | 1(B) |  | 1 | 1 |  |  |
| CC354 | 354 |  |  |  | 3(B);1(H) | 4 | 4 | 4 |  |
|  | 6466 |  |  | 1(H) |  |  | 1 | 1 |  |
|  | 6784 |  |  |  | 1(B) | 1 | 1 | 1 |  |
|  | 7215 |  |  | 1(C) | 1(B);2(C) | 3 | 4 | 4 |  |
|  | 7309 |  |  |  | 1(B) | 1 | 1 | 1 |  |
| CC403 | 933 |  |  |  | 2(C) | 2 | 2 | 2 |  |
| CC42 | 42 |  | 2(H) | 1(B) | 1(B);1(C) | 2 | 3 | 5 |  |
| CC443 | 51 |  | 3(H) |  |  |  | 3 |  |  |
|  | 6391 |  |  |  | 4(B);1(H) | 5 | 5 | 5 |  |
|  | 7208 |  | 1(B) |  |  |  | 1 |  |  |
| CC446 | 446 |  |  |  | 1(H) | 1 | 1 | 1 |  |
|  | 6392 |  |  | 1(H) | 1(B) | 2 | 2 | 1 |  |
| CC45 | 137 |  |  |  | 1(B) | 1 | 1 | 1 |  |
|  | 2067 |  |  |  | 1(WB) | 1 | 1 | 1 |  |
|  | 233 |  |  | 2(B) |  |  | 2 | 2 |  |
|  | 45 |  | 1(H) |  |  |  |  | 1 |  |
|  | 583 |  |  | 1(H) |  |  | 1 | 1 |  |
|  | 7318 |  |  |  | 1(C) | 1 | 1 | 1 |  |
| CC460 | 6467 | 1(H) |  |  |  |  |  |  |  |
|  | 670 |  | 1(H) |  |  |  | 1 |  |  |
| CC464 | 464 |  |  | 5(B);2(H) | 8(B) | 15 | 15 | 8 |  |
| CC48 | 38 |  |  |  | 3(C) | 3 | 3 | 3 |  |
|  | 429 |  | 5(H) | 1(B) |  |  | 6 | 1 |  |
|  | 475 |  |  | 1(B) |  | 1 | 1 |  |  |
|  | 918 |  |  | 1(B);1(H) | 1(H) | 1 | 3 | 3 |  |
| CC52 | 2066 |  | 1(H) | 1(B);2(H) |  |  | 4 | 3 |  |
| CC574 | 305 |  | 2(H) |  |  |  | 2 |  |  |
| CC607 | 607 | 1(H) |  | 2(B) |  |  | 2 | 2 |  |
| CC61 | 61 |  | 1(C) |  | 1(C) | 1 | 1 | 2 |  |
| CC658 | 6468 |  | 1(H) |  |  | 1 |  |  |  |
|  | 658 |  |  | 2(B) | 1(B);1(H) | 3 | 4 | 3 |  |
| CC692 | 692 |  |  | 1(WB) |  |  | 1 | 1 |  |
| CC952 | 2111 |  | 1(WB) | 2(WB) |  |  | 3 | 2 |  |
|  | 2210 | 1(WB) |  |  |  |  |  |  |  |
|  | 2311 |  | 1(WB) |  |  |  | 1 |  |  |
|  | 6228 |  | 3(WB) |  |  |  | 3 |  |  |
|  | 6397 |  | 1(WB) |  |  |  | 1 |  |  |
|  | 6399 |  | 1(WB) |  |  |  | 1 |  |  |
|  | 6402 |  |  | 1(WB) |  |  | 1 | 1 |  |
|  | 6407 | 1(WB) |  |  |  |  |  |  |  |
|  | 6408 |  | 1(WB) |  |  |  | 1 |  |  |
|  | 6434 |  | 1(WB) |  |  |  | 1 |  |  |
| Total | | 6(H);5(WB) | 8(B);55(WB);39(H);2(C) | 48(B);33(WB);6(C);46(H) | 42(B);33(C);10(H);8(WB) | 129 | 312 | 206 | 3 |

H, human cases; C, dairy cattle; B, broiler products; WB, wild birds; CC, clonal complex; ST, sequence type; TET, tetracycline; ERY, erythromycin; CIP, ciprofloxacin; GEN, gentamicin; AXO, ceftriaxone.

**Table S2**

Antimicrobial resistance profiles of the *C. jejuni* clonal complex and sequence types obtained from various sources.

| CC | ST | Antimicrobial resistance profiles of *C.jejuni* | | | | | | | | | |
| --- | --- | --- | --- | --- | --- | --- | --- | --- | --- | --- | --- |
|  |  | 1 | 2 | 3 | 4 | 5 | 6 | 7 | 8 | 9 | 10 |
|  | 1721 |  |  |  |  |  |  |  |  |  |  |
|  | 2117 |  | 1(WB) |  |  |  |  |  |  | 4(C) |  |
|  | 2186 |  | 1(WB) |  |  |  |  |  |  |  |  |
|  | 2217 | 1(C) |  |  |  |  |  |  |  |  |  |
|  | 2743 |  | 1(WB) |  |  |  |  |  |  |  |  |
|  | 2883 |  |  | 1(B) |  |  |  |  |  |  |  |
|  | 3098 |  |  | 1(C) |  |  |  |  |  |  |  |
|  | 3502 |  |  |  |  |  |  |  | 1(B) |  |  |
|  | 3546 |  |  |  |  |  |  |  |  | 1(H) |  |
|  | 3573 |  |  |  |  |  |  |  |  | 1(B) |  |
|  | 3755 |  |  |  |  |  |  |  |  | 1(WB) |  |
|  | 436 |  |  |  |  |  |  | 1(C) |  |  |  |
|  | 448 |  | 1(WB) |  |  |  |  |  |  |  |  |
|  | 4566 |  | 1(WB) |  |  |  |  |  |  |  |  |
|  | 4596 |  |  | 1(WB) |  |  |  |  |  |  |  |
|  | 4800 |  |  | 2(B) |  |  |  |  |  |  |  |
|  | 495 |  | 1(H) |  |  |  |  |  |  |  |  |
|  | 5543 |  |  |  |  |  |  |  |  |  |  |
|  | 5559 |  |  | 1(WB) |  |  |  |  |  |  |  |
|  | 5590 |  |  | 1(C) |  |  |  |  |  |  |  |
|  | 5843 |  |  | 1(WB) |  | 1(WB) |  |  |  |  |  |
|  | 5845 |  |  | 1(WB) |  |  |  |  |  |  |  |
|  | 6383 |  | 1(WB) |  |  |  |  |  |  |  |  |
|  | 6384 |  | 1(WB) |  |  |  |  |  |  |  |  |
|  | 6385 |  | 1(WB) |  |  |  |  |  |  |  |  |
|  | 6386 |  | 3(WB) |  |  |  |  |  |  |  |  |
|  | 6387 |  |  | 2(WB) |  |  |  |  |  |  |  |
|  | 6388 |  | 1(WB) |  |  |  |  |  |  |  |  |
|  | 6389 |  | 1(WB) |  |  |  |  |  |  |  |  |
|  | 6390 |  | 1(WB) |  |  |  |  |  |  |  |  |
|  | 6394 |  | 1(WB) |  |  |  |  |  |  |  |  |
|  | 6395 |  |  | 1(WB) |  |  |  |  |  |  |  |
|  | 6396 |  |  | 2(WB) |  |  |  |  |  |  |  |
|  | 6398 |  |  | 1(WB) |  |  |  |  |  |  |  |
|  | 6400 |  | 1(WB) | 1(WB) |  |  |  |  |  |  |  |
|  | 6401 |  |  | 1(WB) |  |  |  |  |  |  |  |
|  | 6403 |  | 1(WB) |  |  |  |  |  |  |  |  |
|  | 6404 | 1(WB) |  |  |  |  |  |  |  |  |  |
|  | 6405 |  | 1(WB) |  |  |  |  |  |  |  |  |
|  | 6406 | 1(WB) |  |  |  |  |  |  |  |  |  |
|  | 6410 |  | 1(B) | 1(B) |  |  |  |  | 5(B) | 4(B) |  |
|  | 6411 |  |  |  |  |  |  |  | 2(B) | 4(B) |  |
|  | 6412 |  |  |  |  |  |  |  |  | 1(B) |  |
|  | 6415 | 1(WB) |  |  |  |  |  |  |  |  |  |
|  | 6422 |  | 2(WB) |  |  |  |  |  |  |  |  |
|  | 6423 |  |  | 1(WB) |  |  |  |  |  |  |  |
|  | 6425 |  | 1(WB) |  |  |  |  |  |  |  |  |
|  | 6427 |  | 1(WB) |  |  |  |  |  |  |  |  |
|  | 6428 |  |  | 1(WB) |  |  |  |  |  |  |  |
|  | 6429 |  | 1(WB) |  |  |  |  |  |  |  |  |
|  | 6430 |  | 1(WB) |  |  |  |  |  |  |  |  |
|  | 6431 |  | 1(WB) |  |  |  |  |  |  |  |  |
|  | 6432 |  | 1(WB) |  |  |  |  |  |  |  |  |
|  | 6433 |  |  |  |  |  |  |  |  | 1(WB) |  |
|  | 6437 |  | 1(WB) |  |  |  |  |  |  |  |  |
|  | 6438 |  | 1(WB) |  |  |  |  |  |  |  |  |
|  | 6439 |  |  | 1(WB) |  |  |  |  |  |  |  |
|  | 6609 |  |  | 1(WB) |  |  |  |  |  |  |  |
|  | 7207 |  |  |  |  |  |  |  |  | 1(B) |  |
|  | 7209 |  |  |  |  |  |  |  | 1(B) |  |  |
|  | 7213 |  |  |  |  |  |  |  | 1(B0 |  |  |
|  | 7308 |  | 1(B) |  |  |  |  |  |  |  |  |
|  | 7311 | 1(WB) |  |  |  |  |  |  |  |  |  |
|  | 7312 |  |  | 1(WB) |  |  |  |  |  |  |  |
|  | 7315 | 1(WB) |  |  |  |  |  |  |  |  |  |
|  | 905 |  | 1(WB) |  |  |  |  |  |  |  |  |
|  | 992 |  | 1(WB) |  |  |  |  |  |  |  |  |
| CC1034 | 6409 |  |  |  |  |  |  |  |  | 1(B) |  |
| CC1275 | 3925 |  |  |  |  |  |  | 1(WB) |  |  |  |
| CC1287 | 6414 |  | 1(WB) |  |  |  |  |  |  |  |  |
| CC179 | 220 |  | 2(WB) | 3(WB) |  |  |  |  |  |  |  |
|  | 2209 |  | 1(H) |  |  |  |  |  | 1(H) |  |  |
|  | 4447 | 2(WB) |  | 3(WB) |  |  |  |  |  | 3(WB) |  |
|  | 6421 |  | 1(WB) |  |  |  |  |  |  | 1(WB) |  |
|  | 6424 |  | 4(WB) | 4(WB) |  |  |  |  |  |  |  |
|  | 6426 |  | 1(WB) |  |  |  |  |  |  |  |  |
| CC206 | 122 |  |  |  |  |  |  |  |  |  |  |
|  | 227 |  | 5(H) 2(B) |  |  |  |  |  | 1(H) |  |  |
|  | 572 |  |  |  |  |  |  |  |  | 2(C) |  |
| CC21 | 1459 |  | 1(H) |  |  |  |  |  |  |  |  |
|  | 19 |  | 1(H) | 3(H) | 1(H) |  |  |  |  | 1(C) 1(B) |  |
|  | 1943 |  |  | 1(H) |  |  |  |  |  |  |  |
|  | 21 |  |  |  |  |  |  |  | 1(H) 2(B) | 14(C) |  |
|  | 251 | 1(B) |  |  |  |  |  |  |  |  |  |
|  | 376 | 1(H) | 1(B) | 1(B) |  |  |  |  |  | 1(B) |  |
|  | 50 |  | 6(H) | 3(H)3(B) |  |  |  |  | 1(H) | 1(H) |  |
|  | 6393 |  | 1(H) |  |  |  |  |  |  |  |  |
|  | 6436 |  |  | 1(B) |  |  |  |  |  |  |  |
|  | 7211 |  |  | 1(B) |  |  |  |  |  |  |  |
|  | 7216 |  |  | 1(C) |  |  |  |  |  |  |  |
| CC22 | 1947 |  |  |  |  |  |  |  |  |  |  |
|  | 22 | 1(H) |  |  |  |  |  |  |  |  |  |
| CC257 | 257 |  | 2(H) |  |  |  |  | 1(C) | 3(H) | 2(C) |  |
|  | 824 |  |  | 2(H) 1(B) |  |  |  |  |  |  |  |
| CC283 | 6382 |  | 1(H) |  |  |  |  |  |  |  |  |
|  | 7210 |  |  | 1(B) |  |  |  |  |  |  |  |
| CC353 | 3285 |  |  | 1(H) |  |  |  |  |  |  |  |
|  | 353 |  |  | 1(H) |  |  |  |  | 1(H) | 2(B) |  |
|  | 356 |  |  |  |  |  |  |  |  | 1(H) |  |
|  | 5 |  | 2(H) | 18(H)6(B) |  |  |  |  | 1(H) | 3(B) | 1(H) |
|  | 5011 |  | 1(B) |  |  |  |  |  |  |  |  |
|  | 6413 |  |  |  |  |  |  |  |  | 1(B) |  |
|  | 6435 |  |  |  |  |  |  |  | 1(B) |  |  |
|  | 7212 |  |  |  |  |  |  |  | 1(B) |  |  |
| CC354 | 354 |  |  |  |  |  |  |  |  | 1(H0 3(B) |  |
|  | 6466 |  |  | 1(H) |  |  |  |  |  |  |  |
|  | 6784 |  |  |  |  |  |  |  |  | 1(B) |  |
|  | 7215 |  |  | 1(C) |  |  |  |  |  | 2(C) 1(B) |  |
|  | 7309 |  |  |  |  |  |  |  |  | 1(B) |  |
| CC403 | 933 |  |  |  |  |  |  |  |  | 2(C) |  |
| CC42 | 42 | 2(H) |  | 1(B) |  |  |  |  |  | 1(C 1(B) |  |
| CC443 | 51 |  | 3(H) |  |  |  |  |  |  |  |  |
|  | 6391 |  |  |  |  |  |  |  |  | 1(H) 4(B) |  |
|  | 7208 |  | 1(B) |  |  |  |  |  |  |  |  |
| CC446 | 446 |  |  |  |  |  |  |  |  | 1(H) |  |
|  | 6392 |  |  |  |  |  |  |  | 1(H) | 1(B) |  |
| CC45 | 137 |  |  |  |  |  |  |  |  | 1(B) |  |
|  | 2067 |  |  |  |  |  |  |  |  | 1(WB) |  |
|  | 233 |  |  | 2(B) |  |  |  |  |  |  |  |
|  | 45 | 1(H) |  |  |  |  |  |  |  |  |  |
|  | 583 |  |  | 1(H) |  |  |  |  |  |  |  |
|  | 7318 |  |  |  |  |  |  |  |  | 1(C) |  |
| CC460 | 6467 |  |  |  |  |  |  |  |  |  |  |
|  | 670 |  | 1(H) |  |  |  |  |  |  |  |  |
| CC464 | 464 |  |  |  |  |  |  |  | 2(H) 5(B) | 8(B) |  |
| CC48 | 38 |  |  |  |  |  |  |  |  | 3(C) |  |
|  | 429 |  | 5(H) | 1(B) |  |  |  |  |  |  |  |
|  | 475 |  |  |  |  |  |  |  | 1(B) |  |  |
|  | 918 |  |  | 1(H)1(B) |  |  |  |  |  | 1(H) |  |
| CC52 | 2066 |  | 1(H) | 2(H)1(B) |  |  |  |  |  |  |  |
| CC574 | 305 |  | 2(H) |  |  |  |  |  |  |  |  |
| CC607 | 607 |  |  | 2(B) |  |  |  |  |  |  |  |
| CC61 | 61 | 1(C) |  |  |  |  |  |  |  | 1(C) |  |
| CC658 | 6468 |  |  |  |  |  | 1(H) |  |  |  |  |
|  | 658 |  |  | 1(B) |  |  |  |  | 1(B) | 1(H) 1(B) |  |
| CC692 | 692 |  |  | 1(WB) |  |  |  |  |  |  |  |
| CC952 | 2111 |  | 1(WB) | 2(WB) |  |  |  |  |  |  |  |
|  | 2210 |  |  |  |  |  |  |  |  |  |  |
|  | 2311 |  | 1(WB) |  |  |  |  |  |  |  |  |
|  | 6228 |  | 3(WB) |  |  |  |  |  |  |  |  |
|  | 6397 |  | 1(WB) |  |  |  |  |  |  |  |  |
|  | 6399 |  | 1(WB) |  |  |  |  |  |  |  |  |
|  | 6402 |  |  | 1(WB) |  |  |  |  |  |  |  |
|  | 6407 |  |  |  |  |  |  |  |  |  |  |
|  | 6408 |  | 1(WB) |  |  |  |  |  |  |  |  |
|  | 6434 |  | 1(WB) |  |  |  |  |  |  |  |  |

1, AXO ; 2, CIP; 3, CIP+AXO; 4, CIP+AXO+ERY; 5, CIP+ERY; 6, TET; 7, TET+AXO; 8, TET+CIP; 9, TET+CIP+AXO; 10, TET+CIP+ERY.

H, human cases; C, dairy cattle; B, broiler products; WB, wild birds; CC, clonal complex; ST, sequence type; TET, tetracycline; ERY, erythromycin; CIP, ciprofloxacin; GEN, gentamicin; AXO, ceftriaxone.
